# Organic fertilizer substituting 20% chemical N increases wheat productivity and soil fertility but reduces soil nitrate-N residue in drought-prone regions

**DOI:** 10.3389/fpls.2024.1379485

**Published:** 2024-04-23

**Authors:** Jun Zhang, Shuang Li, Peipei Jiang, Rongrong Wang, Jinhua Guo, Huishu Xiao, Jinzhi Wu, Muhammad Shaaban, Youjun Li, Ming Huang

**Affiliations:** ^1^ College of Agriculture, Henan University of Science and Technology, Luoyang, Henan, China; ^2^ College of Biopharmaceutical and Food Engineering, Shangluo University, Shangluo, Shaanxi, China

**Keywords:** dryland, organic fertilizer substitution, grain yield, grain protein, N use efficiency, soil fertility, nitrate-N residue

## Abstract

Organic fertilizer substitution is an effective measure for increasing both the quantity and quality of wheat grain while reducing chemical fertilizer input. However, the effects of reducing nitrogen (N) fertilizer combined with organic fertilizer substitution on grain yield, grain protein content and protein yield, plant N accumulation and translocation, N use efficiency, soil fertility, N apparent surplus and nitrate-N residue in rain-fed drought-prone areas remains limited. In this study, field experiments were conducted over four consecutive seasons (2019-2023) at two sites with four treatments: zero N application (ZN), farmer N application (FN), reduced 20% N of FN (RN), and organic fertilizer substituting 20% N of RN (OSN). The results showed that compared with the ZN treatment, the FN, RN and OSN treatments increased grain yield and its components, grain protein content and protein yield, aboveground N accumulation at the anthesis and maturity stages, pre-anthesis N translocation, post-anthesis N accumulation, N use efficiency, soil fertility. Compared with RN and FN, OSN increased grain yield by 17.12% and 15.03%, grain protein yield by 3.31% and 17.15%, grain N accumulation by 17.78% and 15.58%, and N harvest index by 2.63% and 4.45% averaged across years and sites, respectively. Moreover, OSN increased the contents of organic matter, total N, available P and available K in both 0-20 and 20-40 cm soil layers, decreased N apparent surplus and nitrate-N residue in 0-100 cm, and pH in both 0-20 and 20-40 cm soil layer. Fundamentally, this study suggests that integrating a 20% reduction N from conventional farmer practices with the utilization of organic fertilizer to replace 20% of the chemical N fertilizer (OSN) represents an effective strategy. This approach shows promise in enhancing wheat grain yield, grain protein yield, and N use efficiency. Additionally, it supports the improvement of soil fertility while simultaneously reducing soil nitrate-N residues and the apparent surplus of N in rain-fed drought-prone regions.

## 1 Introduction

Wheat (*Triticum aestivum* L.) is one of the most important staple cereals worldwide, providing approximately 20% of the global calorie and protein supply (Zhong et al., 2019). Therefore, boosting wheat yield and quality plays an important role in food safety. In China, wheat accounted for 20.4% of the total crop area and 24.3% of the total food crop production (National Bureau of Statistics, 2020). Despite this, around 1/3 of wheat production emanates from China’s rain-fed drylands—regions characterized by aridity, semi-aridity, and humid drought-prone (Wu et al., 2023b). In these regions, precipitation is the main water sources for wheat growth, and the seasonal unevenness of rainfall causes the grain production potential to decrease by up to 60-70% (Ren et al., 2010; Zhang et al, 2020); meanwhile, water limitation during wheat growth stages hinders nutrient uptake from the soil by plants, thereby resulting in wheat quality fluctuation including protein content under different rainfall patterns. For example, in the Heyang County, where belongs to typical dryland in China, the wheat protein content in humid year is 8.3% and 5.2% lower than that in dry year and normal year, respectively (Liu et al., 2021). Nevertheless, nutrient shortfalls impose significant constraints of crop production in these regions (Zhang et al., 2023). On the pursuit of the targets for high yield and high quality, the mass even excessive chemical fertilizer was applied to wheat field to minimize the limitation of water and nutrients, which leads to the low efficiency and great environmental risk (Bom et al., 2018; Lu et al., 2022; Nadeem et al., 2022). Therefore, it is imperative to improve fertilization practices to increase grain and protein yield, and resource efficiency and reduce the environmental risk for sustainable wheat production in rain-fed drought-prone areas.

Nitrogen (N), one of the most important macro elements for crop growth and development (Makino, 2011; Jones et al., 2013), plays an important role in uplifting the grain yield and quality wheat (Tilman et al., 2011; McLellan et al., 2018). To boost wheat productivity, high quantities of manufactured N are used in China and throughout the world (Wang et al., 2023). Liu et al. (2018) concluded that a scientific N application was beneficial to root growth, alleviates the negative impact to some extent, and thus promotes water and N uptake by wheat to increase the grain yield in drought-prone areas. However, applying excessive amounts of N fertilizer is not conducive to high-yielding and high-efficient production of wheat (Bappa et al., 2014; Yang et al., 2021). In rain-fed wheat production, the recommended N rate is between 150 and 250 kg ha^-1^, but generally the N rate is more than 225 kg ha^-1^ in farmer production (Zhang et al., 2019; Luo et al., 2020). These long-term improper applications of N fertilizer have caused low N use efficiency and high risk of nitrate-N residue (Zhou et al., 2016; Bom et al., 2018). Thus, fertilization strategies that provide food security and environmental sustainability through optimizing the application rate of chemical N fertilizer are urgently required (Peng et al., 2017; Wang et al., 2020; Ren et al., 2023). Cao et al. (2023) found that 20% reduction of N application had the highest wheat yield in plastic film mulching maize-no-tillage wheat rotation system. While Zhang et al. (2023) concluded that reduced 20% the application rate of chemical N fertilizer did not significantly decrease wheat yield in rice-wheat cropping system. These studies showed that reduced 20% of N fertilizer may maintain or even increase wheat yield, and it is feasible in practice.

Organic fertilizer substitution is an effectively technique to coordinate with soil fertility and crop production (Saudy et al., 2020; Abd-Elrahman et al., 2022). Many studies have shown that organic fertilizer substitution can increase soil organic matter content, promote soil fertility, and improve crop yield. Xia et al. (2017) showed substituting organic fertilizer for chemical fertilizer increased crop productivity by 6.8%. He et al. (2022) found that organic fertilizer substitution increased soil organic matter, alkali-hydrolyzable N, available phosphorus (P) and available potassium (K) in both low fertility soil and high fertility soil. Abbasi and Tahir (2012) concluded that organic fertilizer substituting 25% chemical N fertilizer could ensure wheat yield stability, promote N uptake, and increase N utilization by 20%. Wu et al., 2023a found that organic fertilizer substituting 33% chemical N fertilizer significantly increased wheat protein content and protein yield by 8.0% and 6.3%, respectively. With the advancement of China’s “Zero Growth of Chemical Fertilizer” action in recently years, the organic fertilizer substitution techniques become more widely used in agricultural production (Wang et al., 2021), which is favorable for the local fertilizer management converted towards more sustainable practice. However, the impacts of organic fertilizer substitution varied markedly owing to the great differences of wheat production regions in climate conditions, cropping systems and fertilization methods (Zhang et al., 2015; Lv et al., 2023), thus suitable fertilizer management practices should be further explored and carefully adapted and adopted, taking advantages of local resources (Tan et al., 2013; Liu et al., 2019).

In practice, only a minority of farmers opt to use organic fertilizer for food crops, particularly in dryland wheat production areas. This reluctance can be attributed to several factors. Primarily, the limited availability of organic fertilizer and its relatively high cost mean it is predominantly used for high-profit crops such as organically grown food, vegetables, and fruits, rather than for grain crops (Zhang et al., 2020; Shi and Yang, 2023). Additionally, organic fertilizers often do not show immediate benefits, in contrast to the readily observable impact of chemical fertilizers on crop growth and yield formation (Case et al., 2017; Li et al., 2021). Furthermore, research on organic fertilizer lags behind that of chemical fertilizers, which restricts the application and broader adoption of organic alternatives (Ju and Zhang, 2021; Shi and Yang, 2023). This situation highlights the imperative for more comprehensive studies to examine the effects of substituting organic for chemical fertilizers on winter wheat productivity. Such research would offer robust field demonstrations to encourage farmers in rain-fed, drought-prone areas to adopt organic fertilization methods.

Within these contexts, our study proposed that integrating reduced N of farmer practice with organic fertilizer substituting could optimize N productivity in winter wheat and reduce soil nitrate-N residue. A 4-year experiment was employed in two sites in a semi-humid, drought-prone region. The objectives were to (1) quantify the impact of reduced N of farmer practice, organic fertilizer substituting on wheat yield, protein content and yield and N use efficiency, and (2) evaluate their effects on soil fertility, N apparent surplus and nitrate-N residue, and (3) identify an optimal agronomic strategy that synergizes wheat productivity, soil fertility and nitrate-N residue in these challenging environments.

## 2 Materials and methods

### 2.1 Experimental site description

**Figure 1:**
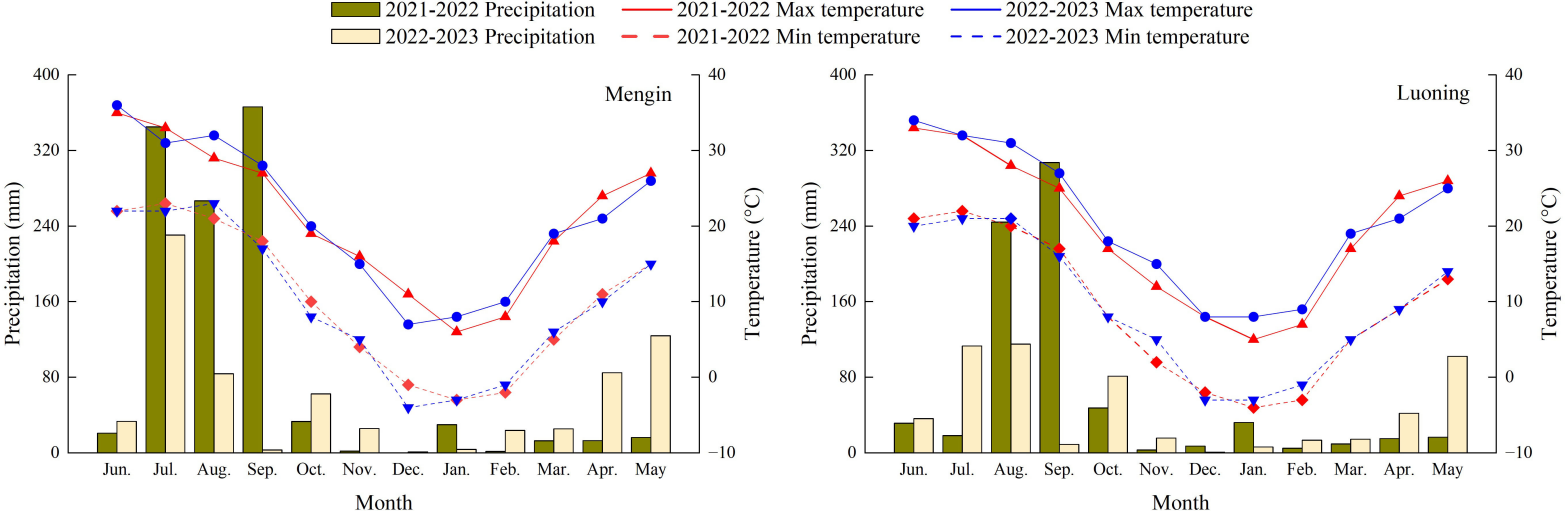
Monthly precipitation during the experiment.

An experiment was performed 4 years from October 2019 to June 2023 in two sites—Mengjin (34°49′N, 112°35′E) and Luoning (34°47′N, 111°71′E) in Luoyang, the typical rain-fed drought-prone area (junction of the Loess Plateau and the Huang-Huai-Hai Plain), China. The altitude of Mengjin site and Luoning site are 262 m and 560 m, respectively. During the present study, the annual average temperature and precipitation of Mengjin site were 14.6°C and 610.2 mm, and that of Luoning site were 13.7°C and 552.9 mm, respectively (**Figure 1**). Winter wheat-summer maize is the typical cropping system in Mengjin and winter wheat-summer fallow in Luoning. The soils of both the two sites are classified as brown soil. At the initiation of the experiment in 2019, the basic soil properties (0-40 cm layer) are listed in **Table 1**.

**Table 1:** Basic soil properties of the top 0-40 cm soil layers.

Site	Soil layer (cm)	Organic matter (g kg^-1^)	Total N (g kg^-1^)	Nitrate-N (mg kg^-1^)	Available P (mg kg^-1^)	Available K (mg kg^-1^)	pH
Mengjin	0-20	14.71	1.17	12.05	19.38	139.74	7.54
	20-40	10.74	0.85	8.99	5.64	106.87	7.41
Luoning	0-20	12.72	0.73	7.83	15.68	197.57	7.26
	20-40	9.56	0.54	5.91	3.15	136.71	7.23

### 2.2 Experimental design and field management

The present study was conducted from October 2021 to June 2023. A completely randomized block design was used, with three replicates for each treatment. The plots were 72.0 m^2^ (10.0 m×7.2 m) and 25.2 m^2^ (7 m×3.6 m) in Mengjin and Luoning, with a 1.2 m wide buffer strip between the plots to avoid effects of nutrients accorded the plots. Four treatments were used: (1) zero N application (ZN); (2) farmer N application (FN) as local farmer practice; (3) reduced N application (RN), 20% reduction of N fertilizer based on FN, and (4) organic fertilizer substituting 20% N of reduced N application (OSN). All treatments received same amount P and K fertilizers (P_2_O_5_ 90 kg ha^-1^, K_2_O 60 kg ha^-1^). The application rate of chemical N in FN was 192 kg ha^-1^ and 172 kg ha^-1^ in Mengjin and Luoning, respectively. In OSN, the application rate of chemical N was 122.9 kg ha^-1^ and 110.1 kg ha^-1^, and the application amount of organic fertilizer was 706.6 kg ha^-1^ and 633.0 kg ha^-1^ in Mengjin and Luoning, respectively. N, P, K fertilizers were urea (N, 46%), triple superphosphate (P_2_O_5_, 12%) and potassium sulfate (K_2_O, 50%), respectively. Organic fertilizer (N, 2.6%; P_2_O_5_, 1.5%; K_2_O, 2.5%; organic matter, 50%; living bacteria count ≥20 million g^-1^) was provided by Luoyang Qihe Ecological Agricultural Technology Co., Ltd. (Luoyang, Henan, China). All fertilizers were broadcasted in the corresponding plots as basal fertilizer. Wheat (*Triticum aestivum* L.) cultivars Zhoumai 27 and Luohan 22 were used in Mengjin and Luoning, respectively. In each growing season, wheat was sown in middle or late October, depending on weather conditions, at a seedling rate of 225 kg ha^-1^, and harvested in late May or early June. Weeds, pests, and diseases were controlled with herbicides and pesticides according to local practices.

### 2.3 Measurements and methods

#### 2.3.1 Grain yield and yield components

At maturity, three quadrats covering 1 m^2^ (1m×1m) were randomly harvested from each plot and threshed after air-drying. Grains from the three quadrats of same plot were mixed and weighed, and then 50 ± 5 g grains were oven-dried at 70°C to constant weight to determine the moisture contents in grain. Grain yield was standardized at a 12.5% moisture content. Before harvest, spike number was counted from the two 1 m double rows of each plot. Grain number per spike was determined by calculating grains of 50 randomly selected spikes from the two 1 m double rows. The 1000-grain weight was calculated by weighing 1000 grains of each sample.

#### 2.3.2 N accumulation, translocation, and distribution

At anthesis, 300 stems of relatively uniform were marked in each plot. After that, the 50 marked stems were manually sampled by cutting aboveground parts at the anthesis and maturity stages, and organs were separated into leaves, stem+sheaths, and spikes (further separated into grain and glume+axis at maturity). All samples were first dried at 105°C for 30 min, and then at 70°C until constant weight to measure the aboveground biomass (kg ha^-1^). N concentration was determined by milling the dried samples, passing through a 0.25mm sieve, and then the total N concentration was digested by H_2_SO_4_-H_2_O_2_ and examined using a high-resolution digital colorimeter auto analyzer 3 (AA3, SEAL Company, Germany). The N accumulation in different organs was N concentration multiplied by the dry weight of the corresponding organ.

The calculation formula was as follows (Yu et al., 2023; Li et al., 2023):

(1) Pre-anthesis N translocation amount (kg ha^-1^) = N accumulation of vegetative organs at anthesis − N accumulation of vegetative organs at maturity;
(2) Contribution rate of pre-anthesis N translocation amount to grain (%) = Pre-anthesis N translocation amount/grain N accumulation at maturity×100;
(3) Post-anthesis N accumulation amount (kg ha^-1^) = Grain N accumulation at maturity − shoot N accumulation at anthesis;
(4) Contribution rate of post-anthesis N accumulation amount to grain (%) = Post-anthesis N accumulation/grain N accumulation at maturity×100;
(5) N harvest index (%) = Grain N accumulation at maturity/Shoot N accumulation at maturity×100.

#### 2.3.3 Grain protein content and protein yield

The protein content and protein yield were calculated as follows (Huang et al., 2021):

(6) Protein content (%) = Grain N concentration×5.7;
(7) Protein yield (kg ha^-1^) = Protein content×grain dry matter amount.

#### 2.3.4 Calculation of N use efficiency and N apparent surplus

The N use efficiency was calculated as follows (Huang et al., 2021; Zhang et al., 2023):

(8) N grain production efficiency (kg kg^-1^) = Grain yield/Shoot N accumulation at maturity×100;
(9) N uptake efficiency (kg kg^-1^) = Shoot N accumulation at maturity/N application rate;
(10) N agronomy efficiency (kg kg^-1^) = (Grain yield under N application treatment − grain yield under ZN treatment)/N application rate;
(11) N recovery efficiency (%) = (Shoot N accumulation amount under N application treatment − Shoot N accumulation amount under ZN treatment)/N application rate×100;
(12) N partial factor productivity (kg kg^-1^) = Grain yield under N application treatment/N application rate.

The N apparent surplus was calculated as follows (Li et al., 2015):

(13) N apparent surplus (kg ha^-1^) = N application rate − Grain N accumulation at maturity.

#### 2.3.5 Soil sampling and analysis

Soil samples were taken 3 days after harvesting in 2023. Five soil subsamples from 0 to 100 cm depth with 20 cm increments were randomly collected from each plot using a soil drill (4.0 cm in diameter). After removing visible roots and stones, the fresh soil subsamples were thoroughly mixed to get a composite soil sample. About 300 g soil was collected for soil fertility analysis and the rest was stored in a freezer at -20°C for nitrate-N residue analysis within 7 days. The sample for soil fertility was air-dried and then passed through 2.0 mm sieve. The determination methods of soil fertility were performed according to Chen et al. (2017). External heating with potassium dichromate was used to determine organic matter. Total N was quantified with Kjeldahl digestion and examined using a high-resolution digital colorimeter auto analyzer 3 (AA3, SEAL Company, Germany). The 0.5 mol L^-1^ NaHCO_3_-molybdeenum blue colorimetric method was used to measure available P. The 1 mol L^-1^ ammonium acetate-flame spectrophotometry method was used to measure available K. The soil pH (1:8 soil: water ratio) was measured using a digital pH meter (PHSJ-4F, Leici Company, China). The nitrate-N residue was quantified with the method described by Dai et al. (2015). Briefly, fresh soil samples weighing 5.0 g were extracted with 50 mL of 1.0 mol L^-1^ KCl after shaking for 1h, and then nitrate-N concentration was measured by a high-resolution digital colorimeter auto analyzer 3 (AA3, SEAL Company, Germany). The nitrate-N residues in each soil layer were calculated as follows:

(14) Nitrate-N residue (kg ha^-1^) = H_i_×D_i_×C_i_×0.1. Where Hi is the soil thickness of the i layer (cm), Di is the soil bulk density (g cm^-3^), Ci is the nitrate-N concentration (mg kg^-1^) in the corresponding soil layer and 0.1 is the conversion coefficient.

### 2.4 Statistical analysis

Data were analyzed using Microsoft Excel 2010 (Microsoft Windows, Redmond, DC, USA) and SPSS 18.0 (IBM, Corp., Chicago, IL, USA). The differences test was performed by the Duncan’s test at a 0.05 probability level. Principal component analysis was performed on the measured indexes using SPSS 18.0 and a graphical presentation was generated using Origin 2024 (Origin Lab Corporation, Northampton, USA).

## 3 Results

### 3.1 Grain yield and yield components

**Table 2** presented year (Y) and treatment (T) extremely significantly but not site (S) affected the grain yield, spike number and grain number per spike, and significantly affected 1000-grain weight, the interaction of S and Y, Y and T, and S, T and Y extremely significantly affected on grain number per spike; while the interaction of S and Y significantly affected on 1000-grain weight. Compared with ZN, FN, RN and OSN significantly increased wheat grain yield, spike number and grain number per spike in both years and sites. Compared with FN, RN did not significantly decrease wheat yield, spike number and grain number per spike except grain number per spike in Mengjin and spike number in Luoning in 2022-2023. Compared with RN and FN, the grain yield, spike number, grain number per spike and 1000-grain weight under OSN increased by 17.12% and 15.03%, 11.11% and 7.21%, 6.45% and 2.95%, 1.41% and 1.03%, respectively, averaged across years and sites.

**Table 2:** Effects of different treatments on wheat yield and its components of winter wheat in 2021-2023.

Site	Year	Treatment	Grain yield/ (kg ha^-1^)	Spike number/ (10^4^ ha^-1^)	Grain number per spike	1000-grain weight (g)
Mengjin	2021-2022	ZN	3820 c	411.3 c	28.5 c	39.2 b
		FN	5450 b	476.9 b	33.6 ab	41.0 a
		RN	5373 b	460.8 b	32.9 b	42.0 a
		OSN	6214 a	516.3 a	34.6 a	42.3 a
	2022-2023	ZN	3634 c	398.1 c	26.8 c	39.8 b
		FN	5170 b	431.7 b	37.2 a	41.7 a
		RN	5088 b	415.6 b	35.5 b	41.4 a
		OSN	6038 a	465.2 a	37.7 a	41.7 a
Luoning	2021-2022	ZN	3540 c	398.1 c	26.8 c	39.6 a
		FN	5101 b	431.7 b	32.5 ab	40.4 a
		RN	5057 b	415.6 b	31.5 b	39.7 a
		OSN	5803 a	465.2 a	33.3 a	40.6 a
	2022-2023	ZN	3472 c	366.0 d	29.9 c	39.3 b
		FN	4985 b	406.9 b	35.8 ab	42.4 a
		RN	4824 b	393.8 c	34.6 b	41.8 ab
		OSN	5761 a	427.3 a	37.6 a	42.6 a
Sources of variance (*F*-value)		Site (S)	22.91	51.63	0.57	0.23
		Year (Y)	19.27**	116.68**	186.97**	6.61*
		Treatment (T)	806.77**	48.84**	26.81**	19.59*
		S×Y	2.21	2.26	16.49**	6.17*
		Y×T	0.76	2.04	11.71**	0.61
		S×T	4.66	0.85	0.66	0.25
		S×T×Y	0.09	2.40	8.07**	1.99

ZN, FN, RN and OSN indicated zero N application, farmer N application, 20% reduction of N fertilizer based on FN and organic fertilizer substituting 20% N of RN, respectively. Different lowercase letters within the same year and the same column indicate significant differences at P<0.05 among treatments. * and ** indicate significant difference at P<0.05 and P<0.01, respectively.

### 3.2 N accumulation

**Figure 2:**
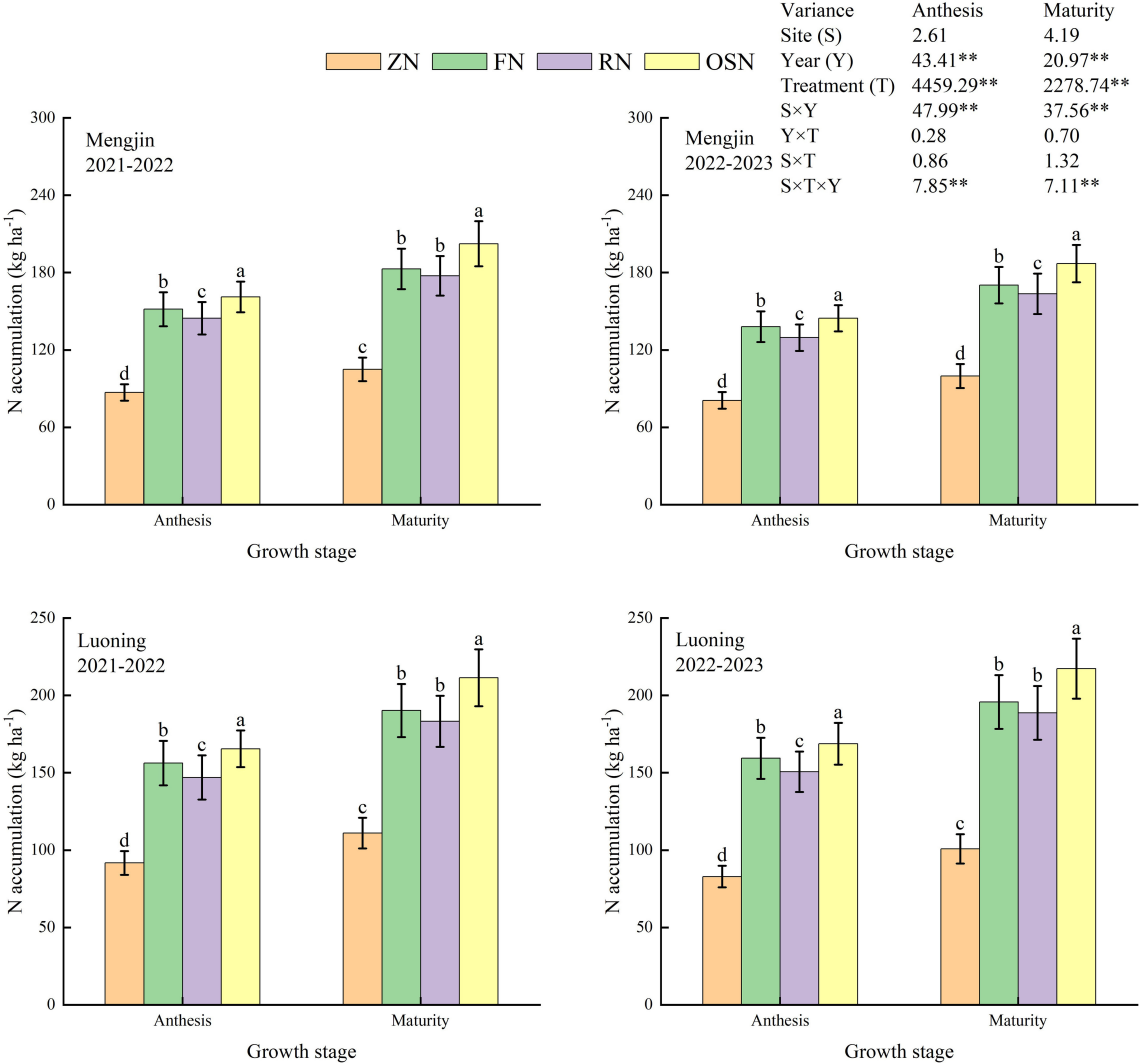
Effects of different treatments on N accumulation at anthesis and maturity of winter wheat in 2021-2023. ZN, FN, RN and OSN indicated zero N application, farmer N application, 20% reduction of N fertilizer based on FN and organic fertilizer substituting 20% N of RN, respectively. Lowercase letters above bars within the same growth stage indicate significant difference among treatments (*P*<0.05). * and ** indicate significant difference at *P*<0.05 and *P*<0.01, respectively.

The year (Y), treatment (T), interaction of S and year (Y), and interaction of S, T and Y extremely significantly affected N accumulation at anthesis and maturity, and the N accumulation at anthesis and maturity showed the same order of ZN<RN<FN<OSN in both years and sites (**Figure 2**). Compared with ZN, FN, RN and OSN increased N accumulation by 72.51%, 63.22% and 82.06% at anthesis and by 72.44%, 66.50% and 90.12% at maturity in Mengjin, respectively; and by 81.35%, 70.97% and 91.99% and by 82.79%, 76.15% and 103.01% in Luoning, averaged across years. These results showed that OSN significantly increased accumulation N at anthesis and maturity, compared with FN and RN. However, RN reduced N accumulation by 5.57% and 3.54% at anthesis and maturity compared with FN, averaged across years and sites.

### 3.3 Pre-anthesis N translocation, post-anthesis N accumulation and N harvest index

As shown in **Table 3**, year (Y), treatment (T) significantly affected the characteristics of pre-anthesis N translocation, post-anthesis N accumulation and N harvest index, and the intersection of S×Y, Y×T, S×T×Y extremely significantly affected N harvest index. Compared with ZN, FN, RN, OSN increased pre-anthesis N translocation amount, post-anthesis N accumulation amount, and contribution rate of post-anthesis N accumulation amount to grain, and thus increased the N accumulation amount in grain, but decreased the N harvest index. Compared with FN and RN, OSN significantly increased N accumulation amount in grain by 14.57% and 16.84% in Mengjin and by 16.59% and 18.70% in Luoning averaged across years, respectively, and thus significantly increased the N harvest index. Compared with FN and RN, OSN increased the N harvest index by 3.91% and 2.30% in Mengjin and by 4.96% and 2.91% in Luoning averaged across years, respectively.

**Table 3:** Effects of different treatments on N translocation, N accumulation and N harvest index of winter wheat in 2021-2023.

Site	Year	Treatment	Pre-anthesis N		Post-anthesis N		N accumulation amount in grain (kg ha^-1^)	N harvest index (%)
			Translocation Amount (kg ha^-1^)	Contribution rate to grain (%)	Accumulation amount (kg ha^-1^)	Contribution rate to grain (%)		
Mengjin	2021-2022	ZN	64.99 d	78.31 a	17.99 c	21.69 c	82.99 c	79.05 a
		FN	99.77 b	76.15 b	31.25 b	23.85 b	131.02 b	71.65 d
		RN	95.64 c	74.38 bc	32.94 b	25.62 ab	128.58 b	72.43 c
		OSN	108.71 a	72.53 c	41.17 a	27.47 a	149.89 a	74.06 b
	2022-2023	ZN	61.01 d	76.36 a	18.89 c	23.64 c	79.90 c	80.09 a
		FN	93.05 b	74.29 ab	32.20 b	25.71 bc	125.25 b	73.58 d
		RN	88.81 c	72.37 bc	33.91 b	27.63 ab	122.71 b	75.08 c
		OSN	101.29 a	70.49 c	42.41 a	29.51 a	143.70 a	76.85 b
Luoning	2021-2022	ZN	68.52 d	78.01 a	19.31 c	21.99 c	87.83 c	79.13 a
		FN	103.69 b	75.35 ab	33.92 b	24.65 bc	137.61 b	72.34 d
		RN	98.96 c	73.17 bc	36.29 b	26.83 ab	135.25 b	73.82 c
		OSN	112.95 a	71.09 c	45.94 a	28.91 a	158.89 a	75.14 b
	2022-2023	ZN	62.78 c	77.81 a	17.90 c	22.19 b	80.68 c	80.03 a
		FN	104.13 b	74.10 ab	36.40 b	25.90 ab	140.53 b	71.76 d
		RN	99.86 b	72.38 b	38.11 b	27.62 a	137.96 b	73.09 c
		OSN	116.87 a	70.64 c	48.57 a	29.36 a	165.44 a	76.12 b
Sources of variance (*F*-value)		Site (S)	4.96	0.08	301.80	0.08	9.54	0.03
		Year (Y)	26.73**	13.30**	3.92	13.29**	6.00*	153.32*
		Treatment (T)	947.43**	2685.30**	472.04**	3167.58**	735.01**	146.43**
		S×Y	24.69**	2.24	0.09	2.25	16.03**	116.37**
		Y×T	1.08	0.02	0.67	0.02	1.90	8.32**
		S×T	1.18	1.05	6.02	1.05	2.06	0.23
		S×T×Y	5.11	0.26	0.56	0.26	5.07**	14.28**

ZN, FN, RN and OSN indicated zero N application, farmer N application, 20% reduction of N fertilizer based on FN and organic fertilizer substituting 20% of RN, respectively. Values within the same year and the same column followed by different lowercase letters indicate significant differences(P<0.05) among treatments. * and ** indicate significant difference at P<0.05 and P<0.01, respectively.

### 3.4 Grain protein content and protein yield

**Figure 3:**
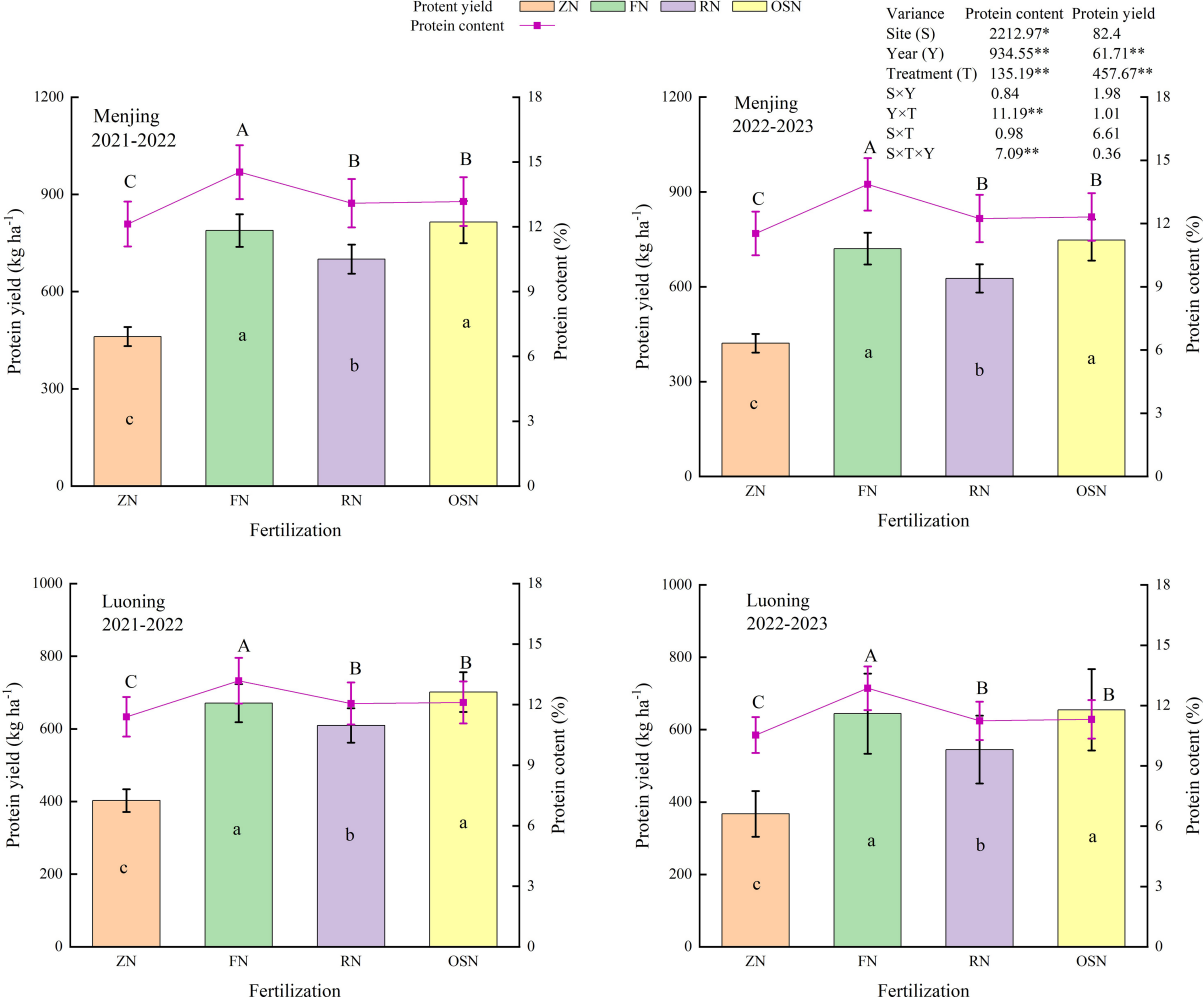
Effects of different treatments on grain protein content and protein yield of winter wheat in 2021-2023. ZN, FN, RN and OSN indicated zero N application, farmer N application, 20% reduction of N fertilizer based on FN and organic fertilizer substituting 20% N of RN, respectively. Different uppercase and lowercase letters indicate that significant difference at *P*<0.05 among treatments, respectively for protein content and protein yield.

**Figure 3** reveals year (Y), treatment (T) extremely significantly affected protein content and protein yield in the both years and sites. Compared with ZN, the protein content under FN, RN and OSN significantly increased by 19.45%, 6.67% and 7.25%, as well as protein yield significantly increased by 71.01%, 50.03% and 76.61% averaged across years and sites, respectively. Compared with FN, RN and OSN significantly decreased the protein content by 11.71% and 10.18%, respectively, while RN significantly decreased protein yield by 12.23%, but OSN increased protein yield by 3.31% averaged across years and sites.

### 3.5 N use efficiency and N apparent surplus

N use efficiency and N apparent surplus was significantly affected by year (Y), Site (S), treatment (T), and their interactions (**Table 4**). Compared with FN and RN, OSN increased N grain production efficiency except Luoning site in 2021-2022, and there were no significant differences between FN and RN. Compared with FN, RN and OSN increased N uptake efficiency, N agronomy efficiency, N recovery efficiency and N partial factor productivity, while RN and OSN decreased N apparent surplus. Compared with RN, OSN significantly increased N uptake efficiency, N agronomy efficiency, N recovery efficiency and N partial factor productivity by 15.22%, 59.47%, 35.93% and 17.12%, averaged across years and sites; and decreased N apparent surplus by 45.82% in Mengjin and 168.43% in Luoning across years. These results showed that OSN could increase winter wheat N use efficiency and decrease N apparent surplus in drought-prone area.

**Table 4:** Effects of different treatments on N use efficiency and N apparent surplus of winter wheat in 2021-2023.

Site	Year	Treatment	N use efficiency					N apparent surplus (kg ha^-1^)
			N grain production efficiency (kg kg^-1^)	N uptake efficiency (kg kg^-1^)	N agronomy efficiency (kg kg^-1^)	N recovery efficiency (%)	N partial factor productivity (kg kg^-1^)	
Mengjin	2021-2022	ZN	36.43 a	—	—	—	—	—
		FN	29.84 c	0.95 b	8.49 b	40.55 b	28.38 c	60.98 a
		RN	30.30 bc	1.03 b	9.03 b	42.17 b	31.24 b	43.42 b
		OSN	30.74 b	1.18 a	13.92 a	56.61 a	36.13 a	22.11 c
	2022-2023	ZN	36.47 a	—	—	—	—	—
		FN	30.40 c	0.89 c	7.99 b	36.73 b	26.93 c	66.75 a
		RN	31.17 bc	0.95 b	8.45 b	37.05 b	29.58 b	49.29 b
		OSN	32.28 b	1.09 a	13.97 a	50.71 a	35.10 a	28.30 c
Luoning	2021-2022	ZN	32.15 a	—	—	—	—	
		FN	27.04 b	1.11 c	9.08 b	46.07 b	29.65 c	27.72 a
		RN	27.79 b	1.21 b	9.98 b	47.51 b	33.27 b	16.75 a
		OSN	27.66 b	1.39 a	14.89 a	66.06 a	38.17 a	-6.89 b
	2022-2023	ZN	34.44 a	—	—	—	—	—
		FN	25.46 c	1.14 c	8.80 b	55.21 b	28.98 c	24.80 a
		RN	25.56 c	1.24 b	9.98 b	57.86 b	31.74 b	14.04 a
		OSN	26.51 b	1.43 a	15.06 a	76.64 a	37.90 a	-13.44 b
Sources of variance (*F*-value)		Site (S)	31.41	17.61	746.89*	3.63	55.25	47.97
		Year (Y)	150	21.41**	2.14	56.73**	17.34**	0.33
		Treatment (T)	54.28*	3415.77**	189.63**	12822.38**	318.87**	1513.30**
		S×Y	24.46**	171.91**	0.01	493.44**	1.10	9.34 **
		Y×T	9.21**	0.34	1.20	0.09	1.07	0.12
		S×T	0.19	22.58*	1.01	11.15	4.27	8.14
		S×T×Y	20.06***	1.04	0.20	2.39	0.16	0.18

ZN, FN, RN and OSN indicated zero N application, farmer N application, 20% reduction of N fertilizer based on FN and organic fertilizer substituting 20% N of RN, respectively. Lowercase letters within the same year and the same column followed by different indicate significant differences (P<0.05) among treatments. * and ** indicate significant difference at P<0.05 and P<0.01, respectively.

### 3.6 Soil fertility

**Figure 4:**
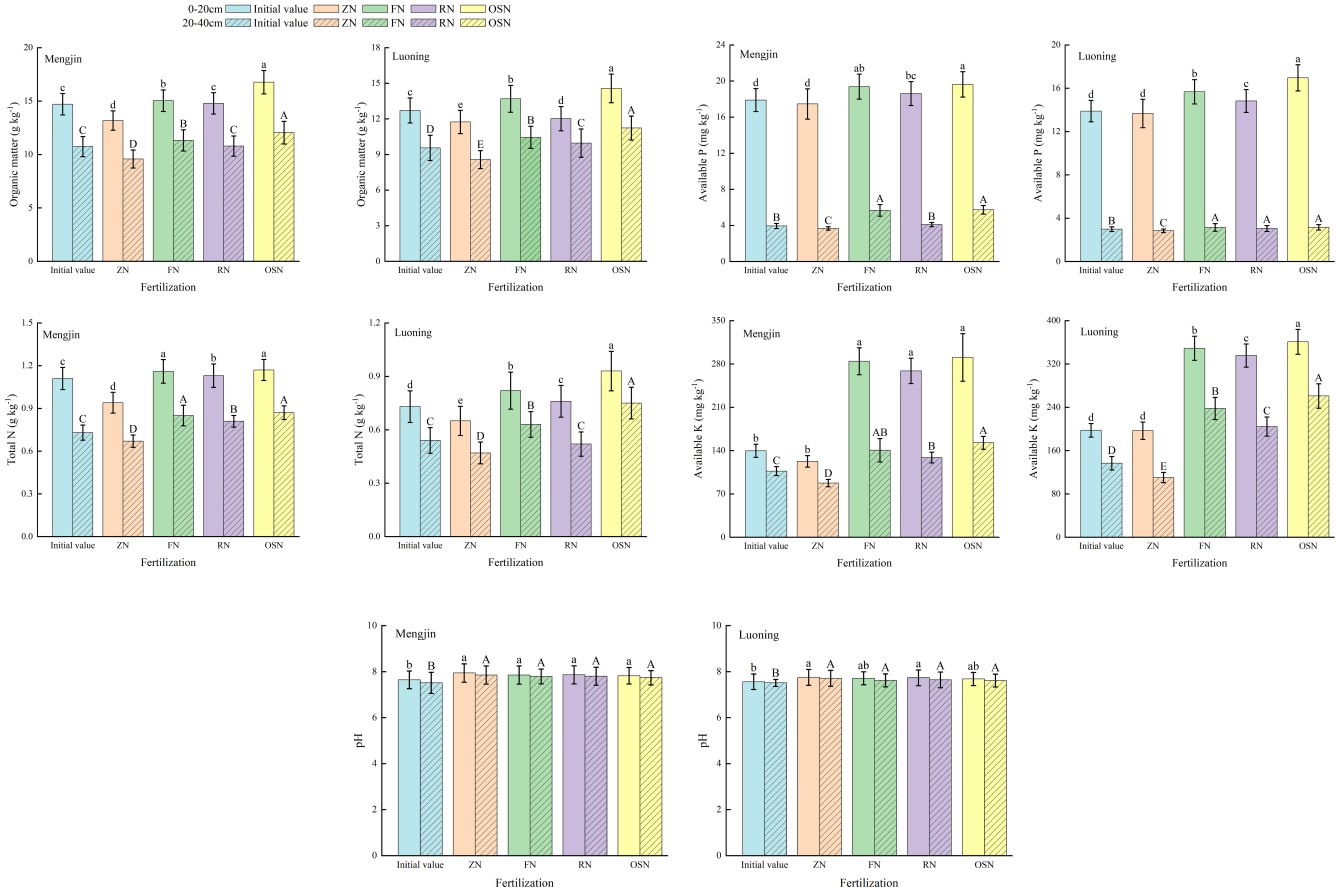
Effects of different treatments on content of organic matter, total N, available P, available K and pH in 0-20 cm and 20-40 cm soil layers at maturity of winter wheat in 2022-2023. ZN, FN, RN and OSN indicated zero N application, farmer N application, 20% reduction of N fertilizer based on FN and organic fertilizer substituting 20% N of RN, respectively. Different lowercase and uppercase letters indicate that significant difference (*P*<0.05) among treatments in 0-20 cm and 20-40 cm soil layer, respectively.

Soil fertility was significantly affected by the 4-year located fertilization in both sites (**Figure 4**). Compared with the initial value, ZN significantly decreased the organic matter and total N but increased pH in both 0-20 cm and 20-40 cm soil layers. The overall trend of organic matter, total N, available P and available K among treatments was ZN<RN<FN<OSN in both soil layers. Compared with FN, RN decreased the contents of organic matter, total N, available P, available K by 6.93%, 4.95%, 4.71% and 4.65%, respectively, in 0-20 cm soil layer, and by 4.69%, 11.08%, 15.02% and 11.21% in 20-40 cm soil layer, averaged across sites. However, OSN increased the contents of organic matter, total N, available P and available K in 0-20 cm soil layer by 17.30%, 12.95%, 9.92% and 7.82% and in 20-40 cm soil layer by 12.17%, 25.82%, 21.85% and 23.03% compared with RN. While compared with RN, OSN decreased pH in 0-20 cm and in 20-40 cm soil layer.

### 3.7 Nitrate-N residue

**Figure 5:**
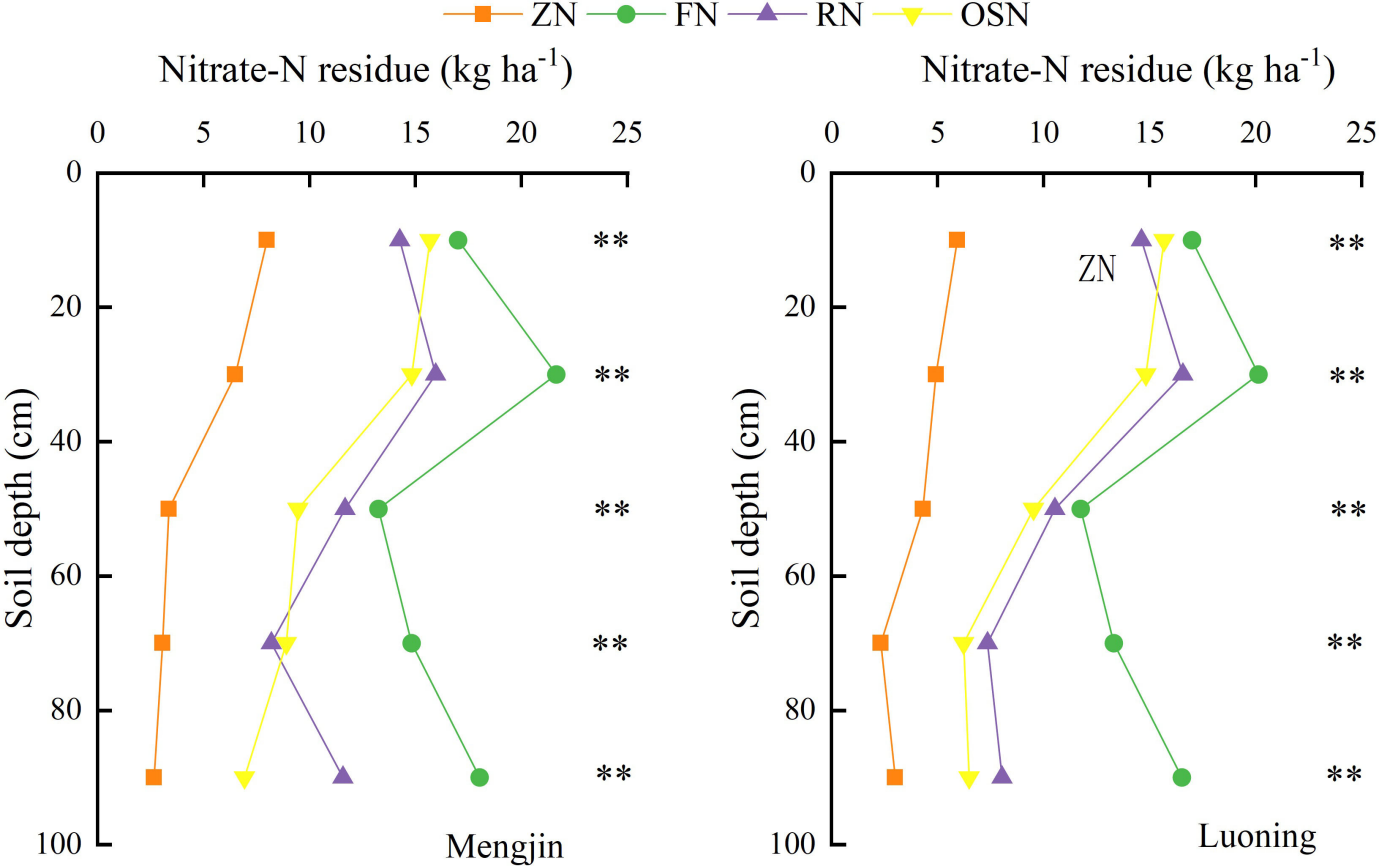
Effects of different treatments on nitrate-N residue in 0-100 cm soil layer at maturity of wheat in 2022-2023 at maturity. ZN, FN, RN and OSN indicated zero N application, farmer N application, 20% reduction of N fertilizer based on FN and organic fertilizer substituting 20% N of RN, respectively. ** indicate that significant differences at *P*<0.01 among treatments within the same soil layer.

As shown in **Figure 5**, the nitrate-N residue in 0-100 cm soil depth were 23.57 kg ha^-1^, 84.69 kg ha^-1^, 61.62 kg ha^-1^ and 55.77 kg ha^-1^ in Mengjin and 20.47 kg ha^-1^, 78.68 kg ha^-1^, 57.09 kg ha^-1^ and 52.73 kg ha^-1^ in Luoning under ZN, OSN, RN and FN, respectively. The ZN showed the lowest nitrate-N residue while FN showed the highest value in each soil layer. Except for 0-20 cm soil layer of both sites and in 60-80 cm soil layer in Mengjin, the nitrate-N residue in 0-100 soil layer of OSN was lower than that of RN, with the average decrease of 20.37% in Mengjin and 12.78% in Luoning across soil layers, indicating that OSN had a beneficial effect on reducing nitrate-N residue.

### 3.8 Comprehensive evaluation

**Figure 6:**
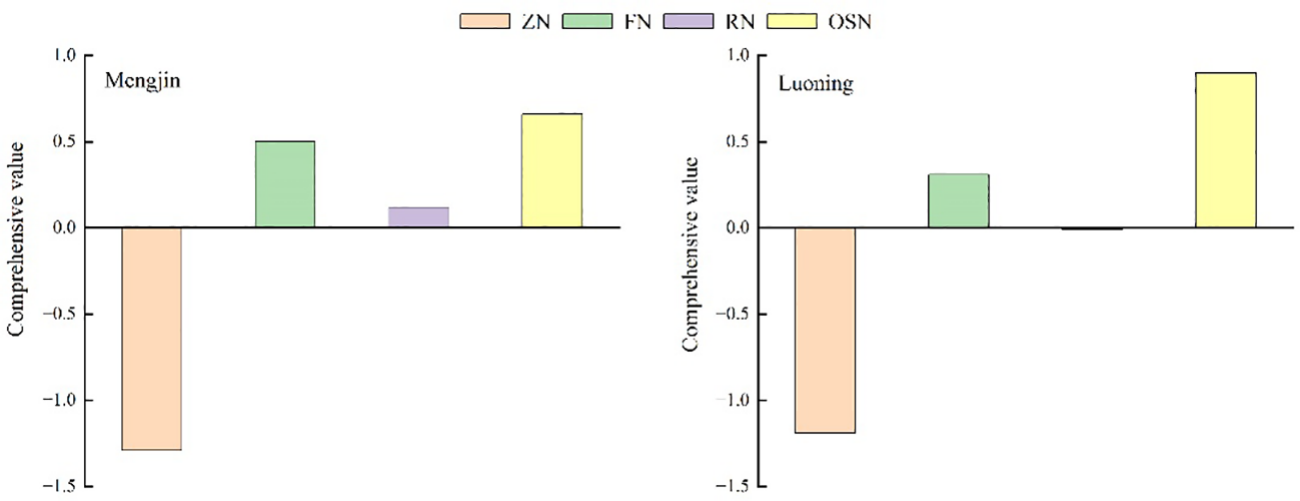
Comprehensive score of different fertilization treatment. ZN, FN, RN and OSN indicated no N application, farmer N application, 20% reduction of N fertilizer based on FN and organic fertilizer substituting 20% N of RN, respectively.

Principal component analysis was used to evaluate the comprehensive effects of different treatments (**Table 5**). The results showed that the N fertilization effects could be explained by the two principal components, with the cumulative contribution rate of 96.82% (Mengjin) and 96.03% (Luoning). The first principal component mainly included grain yield and its components, N translocation and accumulation amount, N accumulation amount in grain, protein content, soil fertility and nitrate-N residue in 0-100 cm soil layer. The second principal component mainly included N harvest index, protein yield and pH in 20-40 cm soil layer. Based on the evaluation formula (y=0.8785C_1_+0.0884C_2_, Mengjin; y=0.8619C_1_+0.0871C_2_, Luoning), the comprehensive score of each treatment could be calculated (**Figure 6**). The comprehensive score ranked as OSN>FN>RN>ZN, meaning OSN had a good comprehensive effect on wheat production, soil fertility and soil nitrate-N residue.

**Table 5:** Score coefficient and contribution rate of principal component.

Indexes	Mengjin		Luoning	
	Component 1 (C_1_)	Component 2 (C_2_)	Component 1 (C_1_)	Component 2 (C_2_)
Grain yield	0.043	-0.074	0.043	0.048
Spike number	0.041	-0.119	0.041	0.136
Grain number per spike	0.043	0.042	0.044	-0.013
1000-grain weight	0.042	-0.028	0.044	-0.035
N accumulation of anthesis	0.043	0.030	0.043	-0.055
N accumulation of maturity	0.044	-0.004	0.044	-0.021
Pre-anthesis N translocation amount	0.044	-0.009	0.044	-0.002
Contribution rate of pre-anthesis N translocation amount	-0.038	0.203	-0.039	-0.113
Post-anthesis N accumulation amount	0.042	-0.116	0.042	0.083
Contribution rate of post-anthesis N accumulation amount	0.038	-0.203	0.039	0.113
N accumulation amount in grain	0.043	-0.046	0.043	0.028
N harvest index	-0.035	-0.238	-0.032	0.303
Protein yield	0.029	0.319	0.028	-0.295
Protein content	0.043	0.044	0.044	-0.051
N grain production efficiency	-0.039	-0.162	-0.040	0.166
Organic matter in 0-20 cm soil layer	0.041	-0.146	0.038	0.125
Organic matter in 20-40 cm soil layer	0.043	-0.055	0.044	0.055
Total N in 0-20 cm soil layer	0.043	0.061	0.042	0.119
Total N in 20-40 cm soil layer	0.043	0.036	0.039	0.163
Available P in 0-20 cm soil layer	0.043	0.025	0.042	0.124
Available P in 20-40 cm soil layer	0.037	0.047	0.043	-0.064
Available K in 0-20 cm soil layer	0.043	0.064	0.043	-0.081
Available K in 20-40 cm soil layer	0.044	-0.002	0.044	-0.017
Nitrate-N residue in 0-100 cm soil layer	-0.044	0.030	-0.041	-0.150
pH in 0-20 cm soil layer	-0.040	0.155	-0.044	0.049
pH in 20-40 cm soil layer	0.034	0.272	0.035	-0.272
Eigenvalue	22.918	2.256	22.760	2.209
Contribution rate (%)	88.146	8.677	87.538	8.496
Cumulative contribution rate (%)	88.146	96.823	87.538	96.034

## 4 Discussion

### 4.1 Effects of organic fertilizer substitution on wheat grain yield, protein content and protein yield

Substituting chemical fertilizers with organic alternatives is a crucial strategy for enhancing crop yields. However, the effectiveness of this approach varies according to the rates of substitution (Shen et al., 2020; He et al., 2022). A meta-analysis revealed that replacing up to 30% of chemical fertilizers with organic ones can lead to an increase in wheat yields. Nevertheless, when the substitution rate surpasses 30%, the beneficial effects on wheat yield become observable only after a decade or more (Li et al., 2021). The present 4-year and 2-site experiment found that the organic fertilizer substituted 20% of chemical N fertilizer (OSN) could optimized winter wheat yield components and improved grain yield. This was consistent with previous the studies in winter wheat-summer maize cropping system (Shen et al., 2020) and wheat-fallow fallow cropping system (He et al., 2022), which showed that the substitution of 20% chemical N fertilizer with organic fertilizer could improve wheat yield. These findings suggest that the substitution with organic fertilizers not only accelerates the release of nutrients from chemical fertilizers (Li et al., 2023) but also leverages the slow-release properties of organic fertilizer nutrients. This dual action enhances nutrient absorption and utilization by wheat throughout its entire growth stage (Yang et al., 2020), ultimately leading to higher yields. Moreover, compared to the FN application, the RN application achieved a relatively stable yield despite a 20% reduction in N. This outcome indicates that current N application rates by farmers could be decreased by at least 20% without compromising yields. The present study also found that, compared with FN and RN, OSN significantly increased spike number and grain number per spike; however, it did not affect the 1000-grain weight. These results demonstrated that OSN increased yield by greatly improving spike number and grain number per spike. The main reason may be the organic fertilizer induces the relatively sufficient nutrients and optimal water supply, boosts the growth development and spike differentiation, and finally improves the spike number and grain number per spike (Zhang et al., 2020).

Protein is an important index to evaluate wheat grain quality. The present study found that, compared with FN, the grain protein content under RN and OSN significantly decreased, which was consistent with the results of Han et al. (2022). These results showed that reduced N fertilizer (20%) had a negative impact on wheat quality in drought-prone area. The reason may be because the amount of N requirement for high-protein was higher than that of high-yield (Ghimire et al., 2021). Conversely, these results indicated that the current N application rate of farmers is reasonable for high-quality production, which was mainly due to the recommended fertilization guidance in China in the past 20 years (Xu et al., 2021). Besides, compared with RN, OSN did not increase the protein content while significantly increased grain yield, meaning that the improvement of OSN on grain yield was prior to that on protein content (Li et al., 2023). Conversely, some studies have found that organic fertilizer substitution increased protein content (Xia et al., 2017). The variability of the effeteness among studies might be related to organic fertilizer type and substitution ratio. Additionally, the significant increase in grain yield helped to reach the highest protein yield under OSN, which indicated that it is feasible for OSN to realize a high protein yield based on the higher yield.

### 4.2 Effects of organic fertilizer substitution on plant N accumulation, translocation, and distribution

Wheat yield and grain protein content are regulated not only by the pre-anthesis stored-N translocation in vegetative organs, but also by post-anthesis N absorption, accumulation, and translocation (Jones et al., 2013; Zhou et al., 2016). In this study, the OSN notably enhanced N accumulation at both anthesis and maturity stages compared to the FN and RN treatments. These results could be related to the ability of OSN to improve precipitation storage effectiveness, mitigate soil water depletion (Zhang et al., 2020), and stimulate plant growth and nutrient content (Thomas et al., 2019). Consequently, this led to the highest N accumulation in the aboveground parts and grains, as shown in **Table 3**. These results are in line with other studies reporting positive effects of organic fertilizer substitution on cereal crops (Saikia et al., 2015; Lv et al., 2023). This could be attributed to OSN offering a better synchronization between N supply and demand, while also contributing to an increase in the size of the grain sink. (Pei et al., 2020). Besides, the source-traits such as aboveground dry matter accumulation, leaf photosynthesis also responded to organic fertilizer substitution (Saikia et al., 2015). In addition, ONS delayed leaf senescence, extended duration of source activity, thus increased the contribution rate of post-anthesis dry matter and N accumulation to grain (Pan et al., 2022). This is maybe explained why OSN improved the characteristics of N accumulation and translocation, and increased N harvest index, finally achieving synergistic improvement of grain yield and protein yield.

### 4.3 Effects of organic fertilizer substitution on N use efficiency and N apparent surplus

N use efficiency is an important index to measure the rationality of fertilization measures (Shen et al., 2020; Yin et al., 2021). In this study, compared with FN and RN, OSN increased N uptake efficiency, N agronomy efficiency, N recovery efficiency and N partial factor productivity increased averaged across years and sites, which was consistence with the results of He et al. (2022) and Lv et al. (2023). These findings could be attributed to several factors: (1) OSN addressed the soil nutrient imbalance caused by the prolonged excessive use of chemical fertilizers (Yang et al., 2018); (2) OSN enhanced soil microbial activity and bettered the micro-ecological environment by elevating the soil’s carbon-to-nitrogen ratio (Liu et al., 2022); (3) The consistent nutrient release from organic fertilizers ensured a steady nutrient availability throughout the growing season (Liu et al., 2021). OSN obtained a reasonable N grain production efficiency, N agronomy efficiency and N partial factor productivity, with the average of 29.30 kg kg^-1^, 14.46 kg kg^-1^ and 36.83 kg kg^-1^, respectively, these indexes fall into the reasonable range of above indexes for 30-60 kg kg^-1^, 10-30 kg kg^-1^ and 40-70 kg kg^-1^, respectively, according to Dobermann (2005) documented. However, the N recovery efficiency in this study was 62.51%, obviously higher than 30-50% reported by Dobermann (2005). This was maybe ascribed the accumulative effect of long-term zero N application under ZN, leaded to lower N accumulation and higher N recovery efficiency.

N apparent surplus is often employed to reflect the N input-output balance of a field, farm or for a specific region (Li et al., 2023; Sapkota and Takele, 2023). Li et al. (2020) reported that the reasonable threshold of N apparent surplus in farmland was 40 kg ha^-1^. In the present study, N apparent surplus under FN and RN were 63.87 kg ha^-1^ and 46.36 kg ha^-1^ in Menjin across years. This indicated that reduced 20% N based on the local farmer practice (FN) still lead to a little higher N apparent surplus in Mengjin. While in Luoning, the N apparent surplus of FN and RN were 26.26 kg ha^-1^ and 25.40 kg ha^-1^ across years, which were below the threshold value at 40 kg ha^-1^. Compared with RN, OSN decreased the N apparent surplus remarkably, under which the average N apparent surplus was 25.21 kg ha^-1^ in Mengjin and -10.17 kg ha^-1^ in Luoning, both of that were lower than the reasonable threshold of 40 kg ha^-1^ reported by Li et al. (2020). These may be due to the OSN induced improvement of soil properties and soil water-holding capability (Yang et al., 2018; Liu et al., 2021), leading to a remarkable increase of wheat growth and shoot N uptake, and finally resulting in an over-consumption of soil N (Yue et al., 2012; Ju and Zhang, 2021). Additionally, this study also found that due to the lower N input and slightly higher grain N output in Luoning, resulting in N apparent surplus was lower than that in Mengjin. Particularly, the N apparent surplus under OSN in Luoning was negative value. Therefore, the over-low N apparent surplus should be pay attention when the OSN technique employed in rain-fed, drought-prone areas.

### 4.4 Effects of organic fertilizer substitution on soil fertility and nitrate-N residue

Evaluation of soil fertility is of great significance to improve farmland quality (Li et al., 2024). In this study, owing to four-year no N application, ZN significantly decreased organic matter, total N, available P and available K except for available P and available K in 0-20 cm soil layer. Also, compared with FN, except for available P and available K in 0-20 cm and available K in Mengjin, the content of organic matter, total N, available P and available K in both 0-20 cm and 20-40 cm soil layer under RN were significantly decreased. These results indicated that ZN and RN went against the maintenance and/or improvement of soil fertility. Favorably, compared with FN, OSN could not only increase the content of organic matter, but also maintained or increased the content of total N, available P and available K in 0-20 cm and 20-40 soil layer. Additionally, available P and available K under ZN showed no significant decrease in 0-20 cm soil layer compared with initial value. This may be because the straw was fully returned to the field, which improved soil phosphorus availability and led to a great number of free potassium ions in straw entered the soil (Zhang et al., 2021; Zhang et al., 2023). Besides, compared with RN, OSN decreased pH in both 0-20 cm and 20-40 cm soil layer. In organic fertilizer, organic acids containing phenolic hydroxyl and carboxyl groups could butter soil acidity (Garcıía-Gil et al., 2004) and decrease soil pH (Zhang et al., 2020), which created a favorable condition for root growth and increased yield (Shen et al., 2020; Zhao et al., 2023).

Nitrate-N residues present in the soil were susceptible to leaching, denitrification and emission if the level exceeds the safe threshold (Wen et al., 2016). Thus, soil nitrate-N residue in 0-100 cm soil layer is usually used to evaluate the sustainability of nutrient management practice (Yang et al., 2020). In this study, the nitrate-N residue under RN in the 0-100 cm soil layer reduced by 22.34 kg ha^-1^ averaged across the sites when compared with FN, indicating that nitrate-N residues could be decreased to a reasonable level by reducing the N rate. Similar results also reported in northern India (Lenka et al., 2013), where the nitrate-N residue in 0-120 cm soil layer could decrease by 62 kg ha^-1^ when the N application rate was reduced from 180 kg ha^-1^ to 120 kg ha^-1^. Besides, the nitrate-N residue of OSN in the 0-100 cm soil layer was reduced by 5.11 kg ha^-1^ (8.61%) averaged cross the sites compared with RN, this was in line with the results of Zhang et al. (2020). The significant improvement of soil physical and chemical properties and microbial activity, which led to effectively utilized soil nitrate-N by crop, maybe explained why less nitrate-N residue was left under OSN (Zhang et al., 2020). In addition, in this study, RN and OSN showed the suitable nitrate-N residues, which were 59.36 kg ha^-1^ and 54.25 kg ha^-1^ across sites, respectively. This residue was around the safe threshold of 50 kg ha^-1^ reported by Zhou and Butterbach-Bahl (2014) and 55 kg ha^-1^ demonstrated by Huang et al. (2017), indicating that RN and OSN can achieve the aim of controlling soil nitrate-N residues in dryland areas.

### 4.5 Solutions to enhance the application of organic fertilizer substitution for wheat production in drought-prone areas

In China, agricultural land is dominated by 200-300 million smallholders (Yin et al., 2021), and around 1/3 of wheat production emanates from China’s rain-fed drought-prone areas (Wu et al., 2023b). However, only a small number of farmlands have been applied organic fertilizer in these areas, so it is necessary to optimize the policy measures to enhance organic fertilizer substitution in wheat production (Case et al., 2017; Li et al., 2021). If subsidies are provided according to organic fertilizer application rather than the wheat planting area, it will improve the enthusiasm of smallholders to apply this suitable fertilizer management practice (Li et al., 2020). Furthermore, enhancing experiments, demonstrations, and training on the substitution of chemical fertilizers with organic alternatives in major wheat-producing regions will also support farmers in adopting this technique. This represents a crucial aspect of our continued efforts in the coming years.

## 5 Conclusion

The results of the present 4-year and 2-site experiment showed that compared with FN and RN, OSN increased grain yield by 17.12% and 15.03%, grain protein yield by 3.31% and 17.15%, grain N accumulation by 17.78% and 15.58%, N harvest index by 2.63% and 4.45% averaged across years and sites, respectively. Besides, OSN significantly increased N use efficiency, as well as soil fertility in both 0-20 cm and 20-40 cm soil layer, while decreased nitrate-N residue in 0-100 cm soil layer by 33.59% and 8.61%, and decreased N apparent surplus by 34.48% and 100.09% compared with FN and RN, respectively. In conclusion, our study revealed that OSN increased grain yield, protein yield and N use efficiency via optimizing wheat N characteristics and soil fertility, and reduced nitrate-N residue and N apparent surplus, thus OSN could be adopted as the suitable fertilization practice to improve grain yield and quality and maintain sustainable agricultural production in rain-fed drought-prone areas.

## Data availability statement

The original contributions presented in the study are included in the article/supplementary material. Further inquiries can be directed to the corresponding authors.

## Author contributions

JZ: Writing – original draft, Writing – review & editing, Conceptualization, Data curation, Formal analysis. SL: Data curation, Formal analysis, Writing – original draft. PJ: Investigation, Writing – original draft, Validation. RW: Writing – original draft, Investigation, Validation. JG: Writing – original draft, Investigation, Validation. HX: Validation, Investigation, Writing – original draft. JW: Project administration, Resources, Writing – review & editing. YL: Writing – review & editing, Funding acquisition, Project administration, Supervision. MS: Writing – review & editing, Validation. MH: Funding acquisition, Project administration, Supervision, Writing – review & editing, Resources.
